# VPS35 D620N mutation impairs neurogenesis and promotes ferroptosis in Parkinson’s disease by using molecular docking, molecular dynamic simulation, and cellular model

**DOI:** 10.3389/fnagi.2025.1692687

**Published:** 2025-11-25

**Authors:** Mei Jiang, Xu Deng, Zijie Qiu, Yuan Fu, Zixiong Qiu, Jiankai Zhang, Hongxia Fu, Jie Li, Yao Luo, Xiaojun Cui

**Affiliations:** 1 The Affiliated Dongguan Songshan Lake Central Hospital, Guangdong Medical University, Dongguan, China; 2 Dongguan Key Laboratory of Stem Cell and Regenerative Tissue Engineering, Department of Human Anatomy, School of Basic Medicine, Dongguan Campus, Guangdong Medical University, Dongguan, China

**Keywords:** VPS35 D620N, Parkinson’s disease, neurogenesis, cell death, ferroptosis

## Abstract

**Backgroud:**

VPS35, a core component of the retromer complex, has been closely associated with neurodegenerative disorders, particularly Parkinson’s disease (PD). The VPS35 D620N mutation has been identified as a pathogenic variant in familial PD. However, the precise mechanisms by which VPS35 and its D620N mutant influence neurogenesis remain poorly understood. This study explores the role of the VPS35 D620N mutation in PD-related neurogenesis.

**Methods and results:**

Protein-protein interaction (PPI) and KEGG pathway analyses identified key regulatory molecules, including TP53, AKT1, and SRC, with the PI3K-Akt signaling pathways emerging as central contributors to mutation-induced neurogenic deficits and ferroptosis in PD. Molecular docking analysis demonstrated strong binding affinities between VPS35 D620N and these hub targets, particularly PI3K. Furthermore, molecular dynamics simulations confirmed the stable interaction between VPS35 D620N and key hub proteins. Immunofluorescence staining revealed that the D620N mutation significantly impaired the neurogenic capacity of neural precursor cells both *in vivo* and *in vitro*, accompanied by increased cell death. Cellular experiments further revealed that the D620N mutation promoted cell death, increased lipid peroxidation and reactive oxygen species (ROS) levels, reduced the expression of ferroptosis-related proteins such as GPX4, and downregulated components of the PI3K-Akt signaling pathway.

**Conclusion:**

This study highlights that the VPS35 D620N mutation may impair neurogenesis through ferroptosis mediated by dysregulation of the PI3K-Akt pathway, offering novel mechanistic insights into its role in PD pathogenesis.

## 1 Introduction

Vacuolar sorting-associated protein 35 (VPS35), as a core component of the retromer complex, plays a crucial role in intracellular protein sorting, autophagy regulation, and the maintenance of synaptic plasticity (Huang et al., 2022; Zavodszky et al., 2014). Abnormalities in its function not only disrupt the lysosomal degradation pathway but also cause an imbalance in synaptic dopamine homeostasis by disturbing the cycling and localization of the dopamine transporter (DAT), ultimately leading to dopaminergic neuronal degeneration—a process that is particularly prominent in Parkinson’s disease (PD) models carrying the VPS35 D620N mutation (Huang et al., 2022; Mohan and Mellick, 2017). For instance, studies in transgenic nematode and mouse models have shown that the VPS35 D620N mutation inhibits the transport of DAT from early endosomes to recycling endosomes by disrupting its interaction with FAM21, the WASH complex, and RAB GTPases, resulting in a reduction of DAT at the cell surface and abnormal accumulation of synaptic dopamine, which in turn induces motor dysfunction and neuronal death (Chen et al., 2019; Huang et al., 2022; Mohan and Mellick, 2017). Additionally, this mutation enhances LRRK2-mediated RAB protein phosphorylation, exacerbates mitochondrial fragmentation and autophagy dysfunction, and further amplifies oxidative stress and neuroinflammatory responses (Mohan and Mellick, 2017; Zavodszky et al., 2014).

Neurogenesis, as a core mechanism of adult central nervous system plasticity, is closely related to the early non-motor symptoms of Parkinson’s disease (PD), such as olfactory dysfunction and cognitive decline. Preclinical studies have shown that the proliferation ability of neural stem cells in the olfactory bulb and hippocampus of PD patients is significantly reduced, and it is negatively correlated with the pathological load of α-synuclein (Cameron and Glover, 2015; Hill et al., 2015; Winner and Winkler, 2015; Sharma et al., 2024). Notably, the VPS35 D620N mutation may interfere with neurogenesis through dual mechanisms: (1) by disrupting WASH complex-dependent membrane transport, it inhibits the secretion of neurotrophic factors (such as BDNF) and the recycling of their receptor TrkB, thereby weakening the differentiation microenvironment of neural stem cells (Zavodszky et al., 2014); (2) through mitochondrial-lysosomal axis dysfunction, it induces apoptosis of neural precursor cells and hinders their differentiation into functional neurons (Chen et al., 2019; Wang et al., 2014). For instance, in a fruit fly model, the expression of VPS35 D620N leads to the late loss of TH-positive dopaminergic neurons, accompanied by decreased motor ability and shortened lifespan, suggesting that neurogenesis defects may precede typical neuronal death (Wang et al., 2014). These findings imply that VPS35 mutations may accelerate the transformation of PD from the subclinical stage to the motor symptom stage by disrupting the dynamic balance of neurogenesis.

Based on previous studies that the VPS35 D620N mutation has been found to regulate the fate of neural stem cells and the role of neurogenesis in the pathogenesis of PD (Qiu et al., 2024; Appel et al., 2018), this study aims to explore the potential mechanism of the VPS35 D620N mutant on neurogenesis through experiment and bioinformatics methods. We will elucidate the regulation of the VPS35 D620N mutation on neurogenesis and assess its potential as a therapeutic target, which provides new insights and references for understanding the mechanisms of PD and developing treatment strategies.

## 2 Materials and methods

### 2.1 Bioinformatics prediction and analysis

#### 2.1.1 Acquisition of the targets of VPS35, PD, and neurogenesis

The keywords “vacuolar sorting associated protein 35 or VPS35,” “Parkinson’s disease or PD,” and “neurogenesis” were searched in the GeneCards database,^1^ and the target sets of VPS35, PD, and neurogenesis were, respectively, sorted out after de-emphasis.

#### 2.1.2 Screening the common targets among VPS35, PD, and neurogenesis

The target sets of VPS35, PD, and neurogenesis were input into the Venny diagram online tool^2^ and, respectively, mapped to obtain the common targets of VPS35, PD, and neurogenesis, and then made a Wayne diagram of the three.

#### 2.1.3 Construct the protein–protein interactions (PPIs) network map of common targets

The common targets obtained in 2.2 were imported into the STRING database,^3^ which limits the study species to “*Homo sapiens*” and sets the value of the lowest interaction domain as the highest confidence. To obtain the target protein interaction information, the minimum interaction domain value was set to the highest confidence value of ≥0.9, and other parameters were kept at the default settings to hide the unconnected nodes. Then, the TSV file was downloaded and imported into Cytoscape software (v3.9.1) for visualization and analysis. Finally, the Network Analyzer function was used to analyze the target sites and get the network analysis results.

#### 2.1.4 Gene ontology (GO) and Kyoto encyclopedia of genes and genomes (KEGG) pathway enrichment analysis

Using the online analytical capabilities of the DAVID database, we import the key targets while selecting “OFFICIAL_GENE_SYMBOL” as the identifier and “*Homo sapiens*” as the species. The list type should be designated as “Gene List.” Following this, submit the data to download the corresponding Gene Ontology (GO) and Kyoto Encyclopedia of Genes and Genomes (KEGG) datasets for further analysis. The downloaded data were subsequently organized into an Excel spreadsheet and imported into Microbiology,^4^ where the results of the enrichment analyses were visually represented through bubble charts and bar graphs.

### 2.2 Molecular docking

To evaluate the interaction strength between VPS35 mutation and relevant target proteins, this study obtained PDB structure files of proteins from the RCSB Protein Data Bank,^5^ and 3D structures of cAMP were downloaded from the PubChem database.^6^ Subsequently, a molecular docking program was conducted on the platform^7^ and the results were visualized with Pymol v3.0 (Singh et al., 2024).

### 2.3 Molecular dynamic simulation (MDS)

Gromacs 2022 was used to conduct 100 ns MDS of the receptors-ligands complexes. The Charmm36 force field was applied to the receptor, and the CGenff force field was used for ligand molecules (Jo et al., 2008). The system was solved using the TIP3P water model with a cubic water box of 1.0 nm (Mark and Nilsson, 2001). The gmx genion tool was used to add ions to the system to achieve electrical neutrality of the system. The particle grid Ewald and Verlet algorithms are used to deal with electrostatic interactions. The system underwent energy optimization before the MDS, including 3,000 steps of steepest descent method optimization and 2000 steps of conjugate gradient method optimization. Finally, MDS trajectories at constant temperature (310 K) and constant pressure (1 bar) for a total of 100 ns, including root mean square deviation (RMSD), root mean square fluctuation (RMSF), radius of gyration (Rg), solvent accessible surface area (SASA), and hydrogen bond count, were analyzed using g-RMSD, g-RMSF, g-Rg, g-SASA, and g-hbond tools, respectively.

### 2.4 Experimental animals

Specific pathogen-free (SPF) grade, clean, and healthy adult VPS35 D620N mutant transgenic (TG) mice, with an average weight of (21 ± 2) grams, were obtained from Shanghai Southern Model Bio-technology Co., Ltd. (Animal Production Licence No. SCXK (Shanghai) 2019–0002). VPS35 wild-type (WT) mice housed in the same cages served as controls. The mice were kept in standard rodent cages under a 12-h light/dark cycle with ad libitum access to food and water. Following a one-week acclimatization period, the experiments commenced. All experimental procedures were followed by protocols approved by the Institutional Animal Care and Use Committee (IACUC) of the Guangdong Medical University (No. GDY2202060).

### 2.5 Animal model treatment and sampling

To minimize the influence of hormones on the experiment, we selected adult male mice for the experiment and randomly divided them into VPS35 wild-type (WT) and VPS35 D620N mutation (TG) groups, with six mice in each group. 10 mg/ml 5-bromodeoxyuridine nucleoside (BrdU) (Roche) was dissolved in 0.9% NaCl solution, then intraperitoneally injected with 50 mg/kg body weight once a day for six consecutive days. Mice were anesthetized by intraperitoneal injection of sodium pentobarbital (60 mg/kg) 24 h after the last injection and then were perfused via the heart with 20 ml of pre-cooled 0.01 M PBS (pH ≈ 7.4), and brain tissue was rapidly removed intact on ice after the liver turned white. In addition, the drop-outs mice during the experiment were euthanized by carbon dioxide (CO_2_) asphyxiation. Adult brain tissues were immersed in 4% paraformaldehyde overnight at 4 °C, fixed and dehydrated with 30% sucrose, and the dehydrated brain tissues were embedded and then coronal sliced with a freezing sectioning machine (Leica) to a thickness of 16 μm, and stored in 0.01 M PBS at 4 °C for later use.

### 2.6 Immunofluorescence staining analysis

The collected brain sections were first washed with 0.01 M phosphate buffer solution (PBS) for 5 min, followed by sealing with 1% bovine serum albumin containing 0.1% Triton X-100 for 30 min before staining with rabbit anti-Ki67 (Abcam, #15580) were incubated at 4 °C overnight. The next day, cells were washed three times with 0.01 M PBS, followed by incubation with fluorescent secondary antibodies Alexa Fluor 555 (Invitrogen, #A31570) and Alexa Fluor 488 (Invitrogen, #A11034) for 1 h protected from light; while the fluorescent nuclear dye DAPI (Sigma-Aldrich, #B2261) was used to counterstaining of cell nuclei. After being washed 3 times with 0.01 M PBS, EdU/BrdU staining was performed with EdU/BrdU Proliferation Analysis Kit (Invitrogen, #10340). Finally, brain sections were washed, dried, and fixed with DAKO fluorescent suspension and observed under a laser confocal microscope (Olympus FV1000). Cell death was detected by using TUNEL staining according to manufacturer’s protocol (Roche, #11684817910).

### 2.7 NPCs isolation, culture, and transfection

Pregnant mice were anesthetized by intraperitoneal injection of sodium pentobarbital (60 mg/kg), then the embryos were removed and put in ice-cold 0.01 M PBS, and the mice were decapitated to extract the brain tissue. Subsequently, these female and dropout mice were euthanized by CO_2_ asphyxiation. The telencephalic lateral ventricle walls of an embryonic 14.5-day C57BL/6 J mouse embryo were dissected using a dissecting microscope. Tissues were triturated and digested with accutase™ Cell Detachment Solution (Stemcell™ Technology, #07922) to isolate enriched NPCs. Subsequently, NPCs were cultured in NPC full-medium containing DMEM/F12 (Gibco) and supplemented with 1% N2, 2% B27 supplement (Gibco), 10 ng/ml human EGF, and 20 ng/ml human FGF (Invitrogen) in a cell incubator at 37 °C until neurospheres were formed. NPCs and N2a cells were cultured in 6-well plates before transfection and were severally transfected with the plasmids, including pLenti6/V5-DEST-GFP, pLenti6/V5-DEST--GFP-VPS35 (WT), and pLenti6/V5-DEST--GFP-VPS35 D620N (D620N), using lipofectamine 2000 (Invitrogen) according to the manufacturer’s protocol. After transfection for 48 h, cells were collected to study the change in neurogenesis.

### 2.8 CCK-8 assay of cell viability

Cell viability was conducted following the instructions of the Cell Counting Kit-8 (Beyotime, #C0038). In brief, cells were seeded in a 96-well plate; 48 h after transfection, 100 ul of cell suspension was prepared in a 96-well plate, and the culture plate was placed in an incubator for 24 h (at 37 °C, 5% CO_2_). Then, 10 ul of CCK-8 solution was added to each well and continued to be incubated in the incubator for 2 h. Finally, the absorbance was measured at 450 nm with a microplate reader.

### 2.9 Lipid peroxidation detection

The relative lipid peroxidation was detected using the Malondialdehyde (MDA) Assay Kit (Beyotime, #S0131M). In brief, 48 h after transfection, cells were collected and then lysed with RIPA buffer. After lysis, cells were centrifuged at 12,000 g for 10 min, and the supernatant was collected for subsequent determination. Subsequently, the protein concentration was determined using the BCA Protein Concentration Assay Kit (Beyotime, #P0009). The TBA storage solution and MDA detection working solution were prepared according to the product instructions. Then, an appropriate amount of standard samples was diluted with distilled water to 1, 2, 5, 10, 20, and 50 μM for subsequent standard curve preparation; 0.1 ml of lysis buffer was added to the centrifuge tube as a blank control, 0.1 ml of the above different concentration standard samples was added to prepare the standard curve, and 0.1 ml of the sample was added for determination; then 0.2 ml of MDA detection working solution was added. After mixing, the sample was heated at 100 °C or in a boiling water bath for 15 min. The sample was cooled to room temperature in the water bath and centrifuged at 1000 g at room temperature for 10 min. Then, 200 μl of the supernatant was added to a 96-well plate, and the absorbance was measured at 532 nm using a microplate reader. Finally, the content of MDA in the cells was calculated.

### 2.10 Reactive oxygen species (ROS) assay

Dihydroethidium (DHE), a superoxide anion fluorescent probe, is bound to DNA by ROS in living cells, and the fluorescence is red. The ROS Assay Kit (Beyotime, #S0033M) contains the fluorescent probe DCFH-DA. ROS can oxidize this probe into green-emitting DCF in cells. DHE and DCFH-DA were diluted with serum-free medium to 5 μM and 10 μM. Remove the cell culture medium and add an appropriate volume of the diluted DCFH-DA. Incubate the cells in a 37 °C cell culture incubator for 20 min. Then, cells were washed three times with 1 × PBS solution to thoroughly remove the DCFH-DA that had not entered the cells. The fluorescence absorbance values were obtained using an enzyme-labeled instrument with an excitation wavelength of 488 nm and an emission wavelength of 525 nm.

### 2.11 Western blotting

Cells were lysed with cold RIPA buffer (25 mM Tris–HCl, pH7.6, 150 mM NaCl, 1% Triton X-100, 1% sodium deoxycholate, 0.1% SDS) supplemented with 1 mM phenylmethylsulfonyl fluoride (PMSF) and a phosphatase inhibitor cocktail (PIC). Samples were then sonicated for 10 s to generate total cell lysates and centrifuged at 12,000 *g* for 20 min to obtain the supernatant. Then, the protein concentration in the different lysates was determined via the BCA assay (Beyotime, #P0009). Lysates were then boiled, and the same amount of protein (20 mg) was loaded and run on 10% SDS-PAGE gels, and the proteins were then transferred to nitrocellulose membranes (0.22 μm), which were blocked with 5% nonfat dry milk in 0.01 TBST at room temperature for 1 h. After blocking, the membrane was cut into strips according to the molecular weight of the target proteins. Each strip was then probed with a specific primary antibody to detect all samples (control, VPS35 WT, and VPS35 D620N). Then, the same set of samples was re-probed with a GAPDH antibody to obtain loading controls, ensuring that the GAPDH signal used for normalization of each target protein originated from the same protein sample loaded on the same membrane. Subsequently, the membrane was incubated with primary antibodies (1:3000) directed against target proteins overnight at 4 °C. The primary antibodies applied were as follows: anti-VPS35 (Abcam, #ab157220), anti-GPX4 (Santa Cruz, sc-166570), anti-GAPDH (Proteintech, #60004-1-Ig), anti-Akt (CST, #9272), anti-p-Akt-Ser473 (CST, #9271), anti-Bcl-2 (Abcam, #ab182858), anti-Bax (Abcam, #ab32503), anti-PI3K (CUSABIO, #CSB-PA854108LA01HU), anti-p-PI3K (Abmart, #TA3242), anti-ERK1/2 (Abmart, #T40071), and anti-p-ERK1/2 (Abmart, #TA1015). Then, membranes were incubated with anti-mouse or anti-rabbit horseradish peroxidase-conjugated secondary antibodies (1:5000, Beyotime, #A0350 and A0352) for 1 h. The final detection of immunoreactive bands was developed using an enhanced chemiluminescent Western blot system (ECL) with exposure to a chemiluminescence imaging system(UVP). The immunoblotting signal intensity was measured using ImageJ 64.

### 2.12 Statistical analysis

The experimental data were analyzed using the statistical software SPSS 20.0. Measurements were expressed as Mean±Standard Error of Mean (Mean±SEM). The differences were analyzed using a two-tailed Student’s t-test and variance ANOVA followed by Tukey’s *post hoc* test. *p*-value was defined at *p < 0.05, **p < 0.01, ***p < 0.001.

## 3 Results

### 3.1 The potential targets of VPS35 and PD neurogenesis have a close interaction

**Figure 1:**
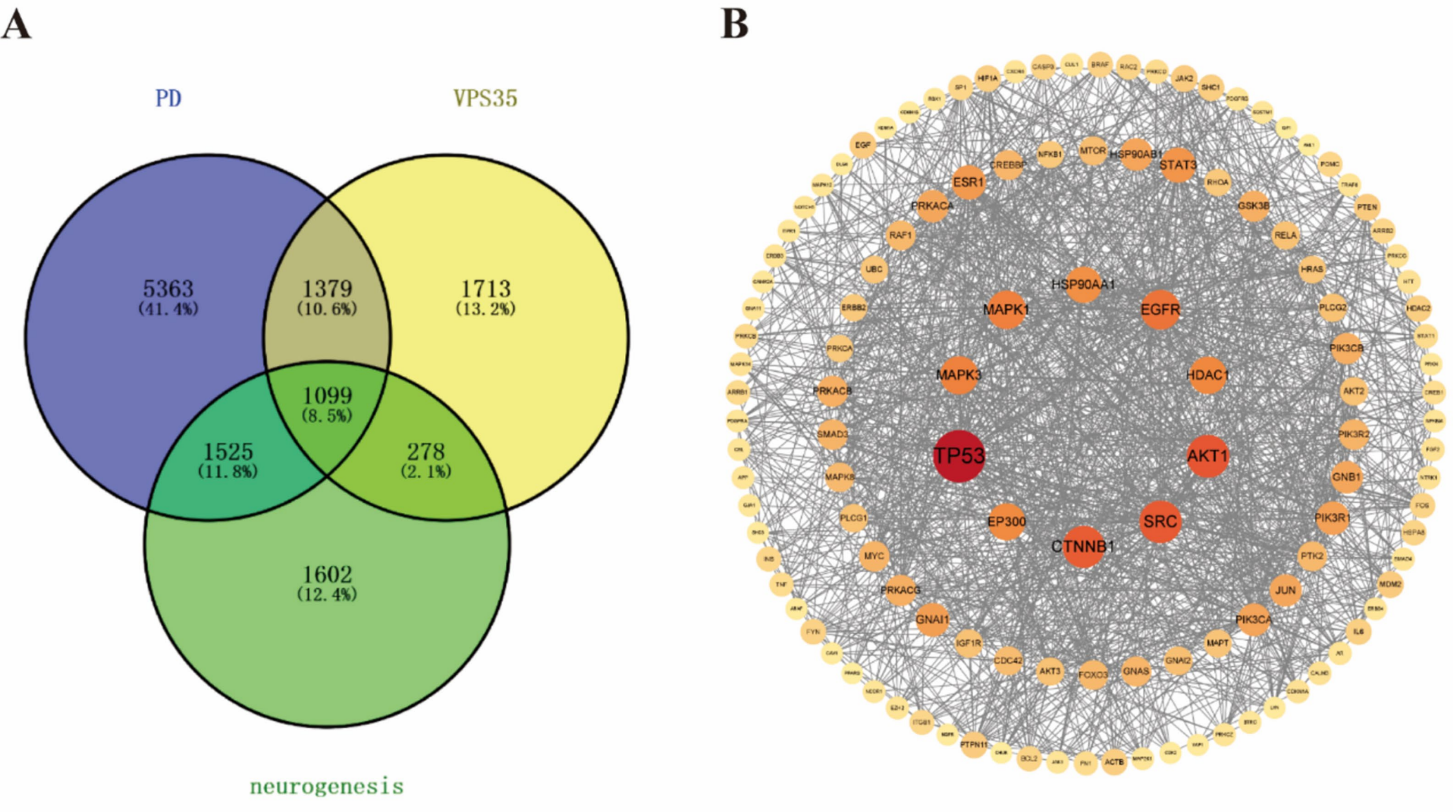
The screening of key targets and PPI analysis. **(A)** Venn diagram of intersecting targets of VPS35, PD, and neurogenesis. **(B)** Co-interacting target PPI network graph. This network graph only shows key targets with a metric value ≥ 20; the darker the node color, the larger the circle, the larger the font, the more important the key target, and the border line represents the interconnections between the targets.

The underlying molecular mechanism by which the VPS35 D620N mutation regulates neurogenesis remains unclear. To elucidate its molecular mechanism on neurogenesis, we anlayzed the respective targets of VPS35 and neurogenesis by using a bioinformatics method. This study obtained 4,469 target genes of VPS35, 4,505 genes of neurogenesis, and 9,366 genes of PD from the GeneCards database. Then, we conducted an intersection analysis of these targets by applying the VENNY online software tool and discovered a significant overlap with 1,099 common genes among VPS35, neurogenesis, and PD (Figure 1A). Subsequently, PPI was performed to explore the interaction of common targets. Data showed that common targets exist a close interconnections and selected the top 10 as the hub targets, including TP53, AKT1, SRC, CTNNB1, EGFR, MAPK1, MAPK3, HDAC1, EP300, and HSP90AA1 (Figure 1B). The detailed information on the hub genes is presented in Table 1, in which the degree value indicates the frequency of interactions between target proteins and serves as a metric for connectivity within the PPI network. A higher degree value shows a more central and influential position in this network of molecular interactions. Targets interact with each other to participate in various aspects of life processes, such as biological signal transmission, gene expression regulation, energy and material metabolism, and cell cycle regulation, which is crucial to understanding the pathogenesis of diseases (Calabrese et al., 2022; Manipur et al., 2023).

**Table 1:** The topological analysis of key targets.

Protein name	Average shortest path length	Betweenness centrality	Closeness centrality	Clustering coefficient	Degree
TP53	2.601511879	0.085800192	0.38439186	0.11053	104
AKT1	2.580993521	0.051833744	0.3874477	0.150516	78
SRC	2.563714903	0.067052524	0.39005897	0.177033	77
CTNNB1	2.688984881	0.06203437	0.37188755	0.116491	76
EGFR	2.653347732	0.053847633	0.37688238	0.163257	69
MAPK1	2.69762419	0.024588011	0.37069656	0.167366	66
MAPK3	2.721382289	0.024735558	0.36746032	0.158654	65
HDAC1	2.976241901	0.027279535	0.33599419	0.162826	63
EP300	2.771058315	0.024010412	0.36087295	0.171867	62
HSP90AA1	2.679265659	0.039093057	0.3732366	0.149718	60

### 3.2 Common targets involve several signaling pathways

**Figure 2:**
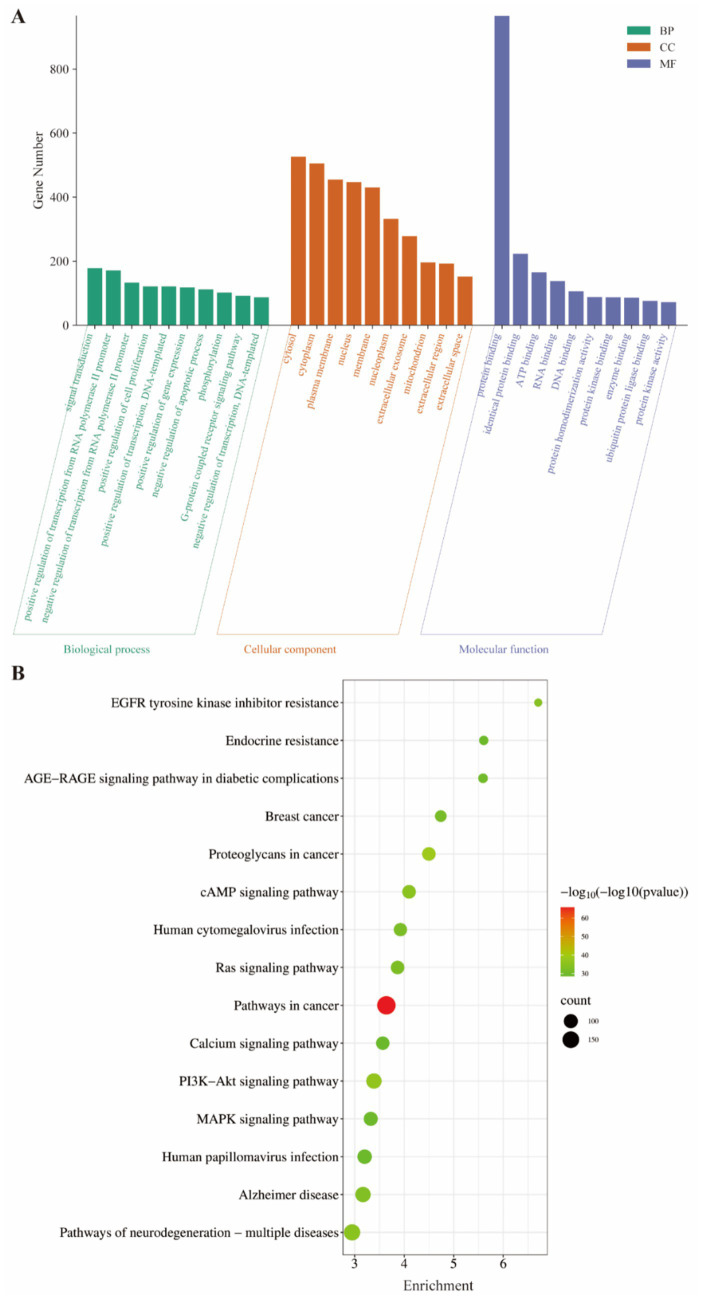
The GO and KEGG enrichment analysis of common targets. **(A)** The top 10 items of GO enrichment analysis bar chart were presented according to the FDR values from smallest to largest, in which the longer length of the bars indicates a higher number of targets. **(B)** The top 15 items of KEGG enrichment analysis were shown according to the FDR values from small to large, where the darker the color and the larger the bubble means the higher the enrichment degree.

GO and KEGG enrichment analysis typically include functional annotation of gene or protein lists, pathway analysis, and disease associations to reveal potential biological significance (Chen et al., 2017). The results of the GO enrichment analysis of common targets indicated that the biological processes primarily revolve around signal transduction and the positive regulation of transcription from RNA polymerase II promoter. Cellular component analysis found a predominant localization within the cytoplasm, plasma membrane, and nucleus. Molecular functions are mainly reflected in the protein binding and ATP binding (Figure 2A). Additionally, KEGG enrichment analysis revealed a significant clustering of these targets within crucial pathways, such as the PI3K-Akt, MAPK, Ras, and cAMP signaling pathways (Figure 2B).

### 3.3 VPS35 exhibits robust interplay with crucial targets and pathways

**Figure 3:**
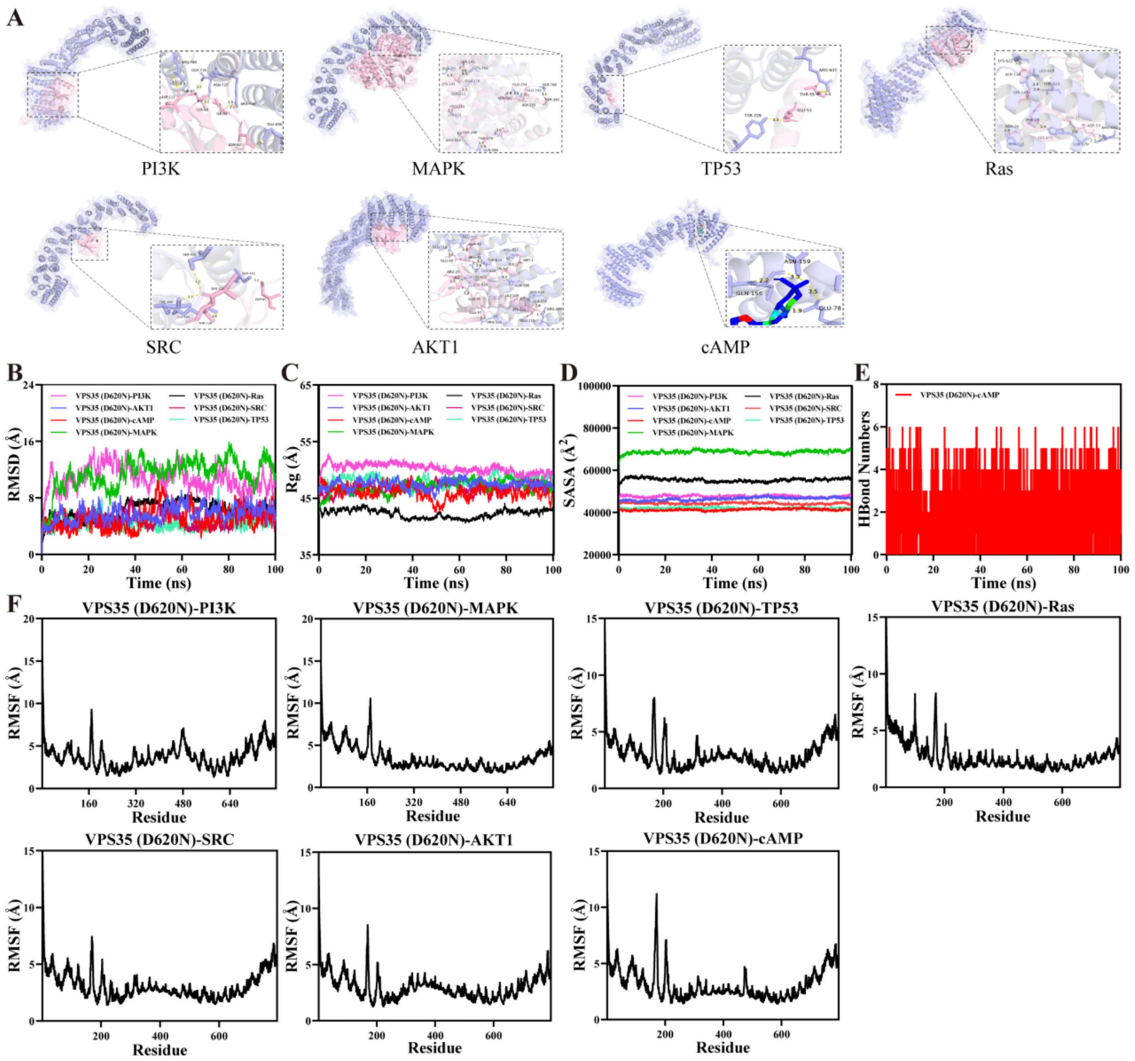
The interaction diagram of key targets by using molecular docking and molecular dynamics simulations. **(A)** The robust binding between the VPS35 D620N mutation and hub genes is shown. It is a widely held scientific tenet that an interaction with binding energy less than −4.25 kcal/mol is indicative of a significant binding affinity between small ligand molecules and their receptor proteins. The binding energy in our study, which is less than −4.25 kcal/mol, suggests an enhanced level of binding activity between the two entities. **(B)** Time-dependent RMSD values of protein–ligand complexes. **(C)** Time-dependent Rg values of protein–ligand complexes. **(D)** Time-dependent SASA values of protein–ligand complexes. **(E)** Time-dependent HBonds values of protein–ligand complexes. **(F)** RMSF values of protein–ligand complexes.

To verify the degree of binding of VPS35 D620N to hub targets, we used the molecular docking technique, which is an important tool for drug design and can be used for large-scale virtual screening (Crampon et al., 2022). The hub targets, for example, PI3K, MAPK, and TP53, demonstrate a robust interaction with VPS35 mutation and values falling below the threshold of −5.0 kcal/mol, which is consistent with the results of KEGG analysis (Figure 3A). The detailed binding energies of interaction are displayed in Table 2.

**Table 2:** The binding energy of molecular docking.

Receptor protein	Ligand molecules	Binding energy (kcal/mol)
VPS35	PI3K	−13.3
	MAPK	−12.5
	TP53	−10.7
	cAMP	−6.4
	SRC	−5.0
	Ras	−5.0
	AKT1	−4.8

MDS was used to further validate the binding of the hub genes. The Root Mean Square Deviation (RMSD) is a reliable indicator of the stability of the protein-ligand complex and reflects the degree of deviation of atomic positions from their initial states. A smaller deviation indicates greater conformational stability. Consequently, RMSD was used to evaluate the equilibrium of the simulation system. MDS results found that VPS35 WT exhibits greater stability and stronger binding affinity to the target. Key indicators such as lower RMSD, reduced bound pocket fluctuations, and more favorable calculation of bound free energy all unanimously support this conclusion (Supplementary Figure 1). The complex of VPS35 D620N mutation with PI3K, MAPK, TP53, Ras, SRC, AKT1, and cAMP exhibited slight fluctuations during the 100 ns simulation period (Figure 3B). Thus, the binding of various ligands to the VPS35 D620N mutation resulted in minor conformational changes. Notably, the VPS35 (D620N)-Ras complex reached equilibrium by the end of the simulation, displaying the lowest RMSD value (6.6 Å), indicating that the VPS35 (D620N)-Ras interaction is characterized by relatively higher stability. Further analysis revealed that the Radius of Gyration (Rg) and Solvent Accessible Surface Area (SASA) of the above seven groups of ligand-receptor complexes exhibited slight oscillations over the course of the simulation (Figures 3C,D). This suggests that although the complexes underwent slight conformational changes during the simulation, they remained overall stable throughout the process. The Root Mean Square Fluctuation (RMSF) indicates the flexibility of amino acid residues in the protein. The number of hydrogen bonds between molecules, with more hydrogen bonds usually enhancing the binding strength and stability of the complex. As shown in Figures 3E,F, the RMSF values of the above seven groups of ligand-receptor complexes were relatively low (mostly below 5 Å), suggesting that the complexes of VPS35 D620N and key targets exhibited low flexibility and high stability.

### 3.4 VPS35 D620N mutation inhibits the proliferation and promotes the cell death of neural progenitor cells

**Figure 4:**
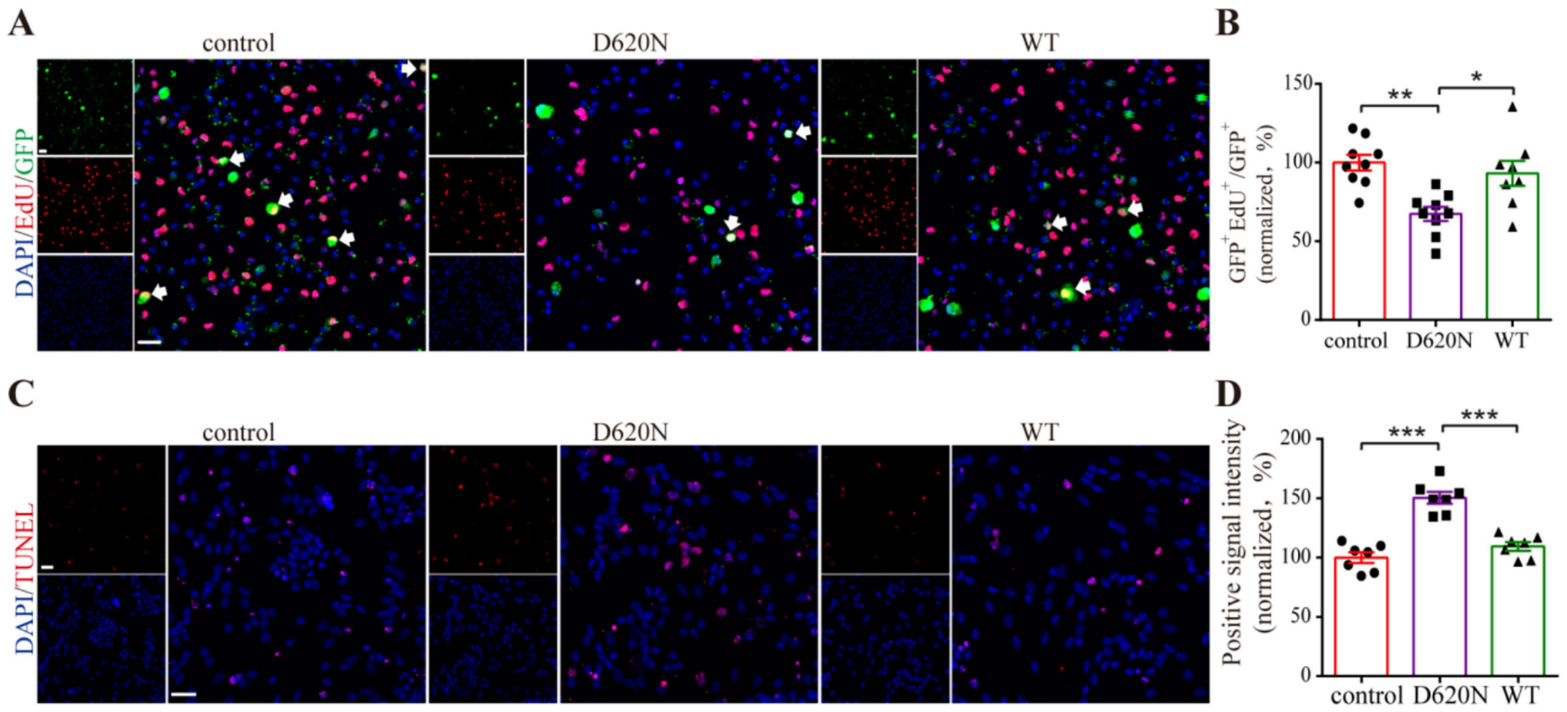
The effect of the VPS35 D620N mutation on NPCs neurogenesis *in vitro*. **(A)** Immunofluorescence staining shows the proliferation markers EdU (red) and GFP (green) in three groups of NPCs, respectively, transfected with empty vector (control), VPS35 wild-type (WT), and VPS35 D620N mutant (D620N) plasmids. The arrow indicates double-labeled positive cells. **(C)** Representative pictures of three groups were detected by TUNEL staining, in which red signals indicate positive cells. Nucleus was stained by DAPI (blue). **(B,D)** Statistic analysis was performed by using One-way ANOVA followed by Tukey’s *post hoc*. The scales are 50 μm **(A)** and 20 μm **(C)**. Arrows show the double-positive cells. Values are shown as the mean ± SEM (*n* ≥ 4), *p < 0.05, **p < 0.01, ***p < 0.001.

Growing evidence found that the VPS35 D620N mutation influenced both neuronal development and synaptic function by suppressing the process of neurogenesis, which caused the progression of PD (Jiang et al., 2021; Qiu et al., 2024). To investigate the impact of the VPS35 D620N mutation on neurogenesis, we first evaluated its effects on neural progenitor cells (NPCs) *in vitro*. Immunofluorescence staining data revealed that the proportion of EdU-positive proliferating NPCs was significantly reduced in the D620N group compared to both the control and WT groups. Notably, no statistically significant difference in EdU incorporation was observed between the WT and control groups (Figures 4A,B). Furthermore, TUNEL staining demonstrated a marked increase in cell death in the D620N-transfected NPCs relative to controls and WT-treated cells (Figures 4C,D). These results collectively demonstrate that the VPS35 D620N mutation suppresses NPC proliferation while promoting apoptosis, suggesting a dual detrimental effect on neurogenic capacity.

### 3.5 VPS35 mutation impairs the adult hippocampal proliferation and cell death

**Figure 5:**
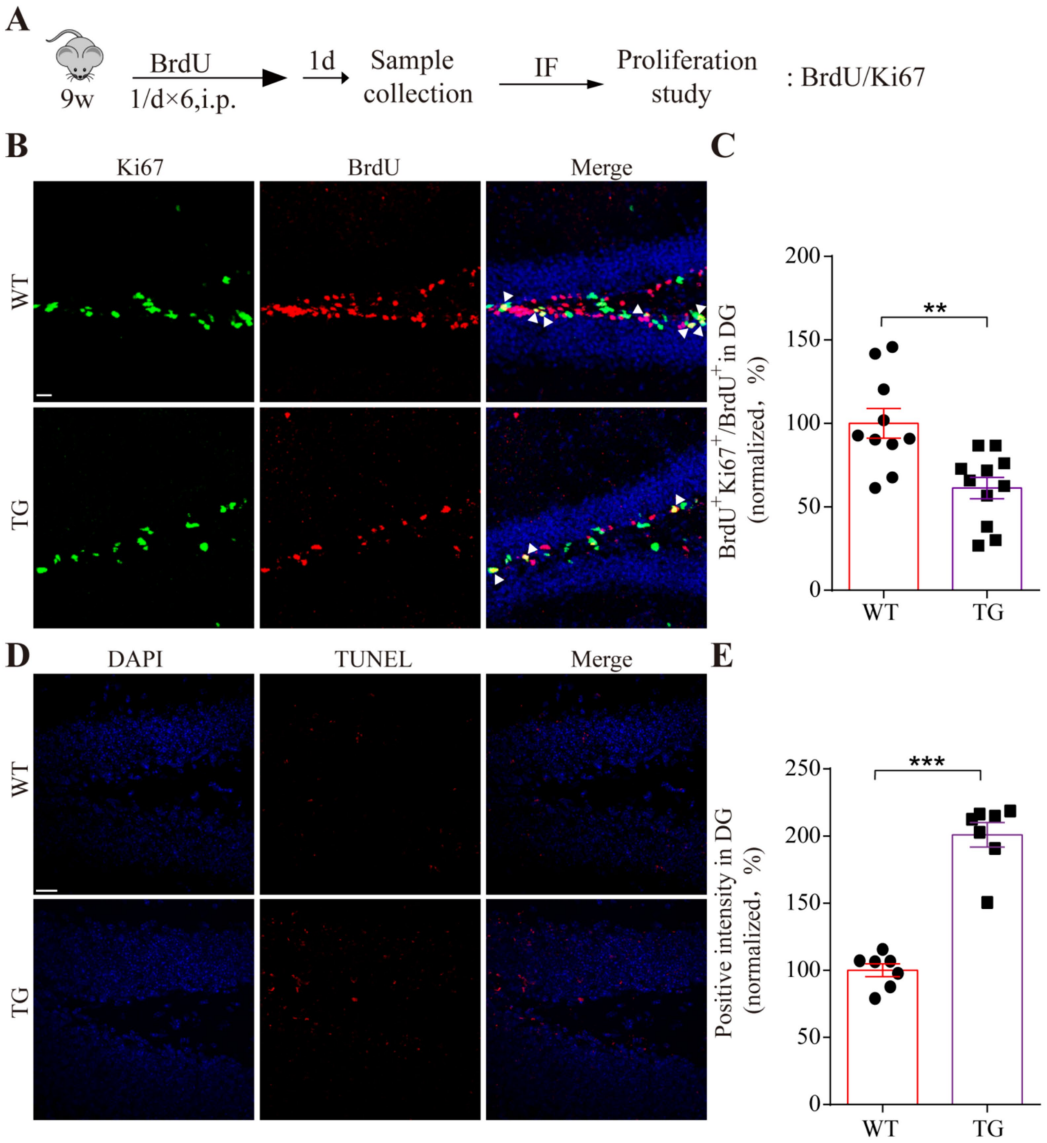
The effect of VPS35 D620N mutation on adult neurogenesis. **(A)** Schematic illustration of BrdU injection in proliferating adult mice. **(B)** Immunofluorescence staining shows the proliferation markers BrdU (red) and Ki67 (green) in the hippocampal DG of VPS35 WT and D620N mice. The triangles represent double-labeled positive cells. **(D)** Representative pictures of two groups were detected by TUNEL staining, in which red signals are positive cells. Nucleus was stained by DAPI (blue). **(C,E)** Statistical analysis was performed by using a two-tailed Student’s *t*-test. The scales of B and D are 20 μm. Arrows show the double-positive cells. Triangles indicate the double-positive cells. Values are shown as the mean ± SEM (*n* ≥ 4), **p < 0.01, ***p < 0.001, two-tailed Student’s *t*-test. DG: dentate gyrus.

Interestingly, it has been reported that the adult VPS35 mutant mouse model impairs neurogenesis by interacting with amyloid precursor protein (APP) (Jiang et al., 2021). To further elucidate the regulatory role of VPS35 mutation on adult neurogenesis, 9-week-old WT and TG mice were intraperitoneally injected with the proliferative marker BrdU (Figure 5A). The immunofluorescence staining results revealed a reduction in the number of BrdU-positive (BrdU^+^) and Ki67^+^ labeled cells within the dentate gyrus (DG) of the TG mice compared to WT mice. In addition, the ratio of double-labeled Ki67^+^ and BrdU^+^ cells to the total BrdU^+^ cells (Ki67^+^ BrdU^+^/BrdU^+^) in the TG adult hippocampal DG was significantly less than that of WT mice (Figures 5B,C). Additionally, consistent with *in vitro* findings, TUNEL staining demonstrated that the cell death within the DG of TG mice has a significant increase compared to the WT group (Figures 5D,E). These results collectively suggest that the VPS35 D620N mutation disrupts adult hippocampal neurogenesis by suppressing progenitor cell proliferation and promoting cell death in the DG microenvironment.

### 3.6 VPS35 D620N impairs cell viability and induces oxidative stress in N2a cells

**Figure 6:**
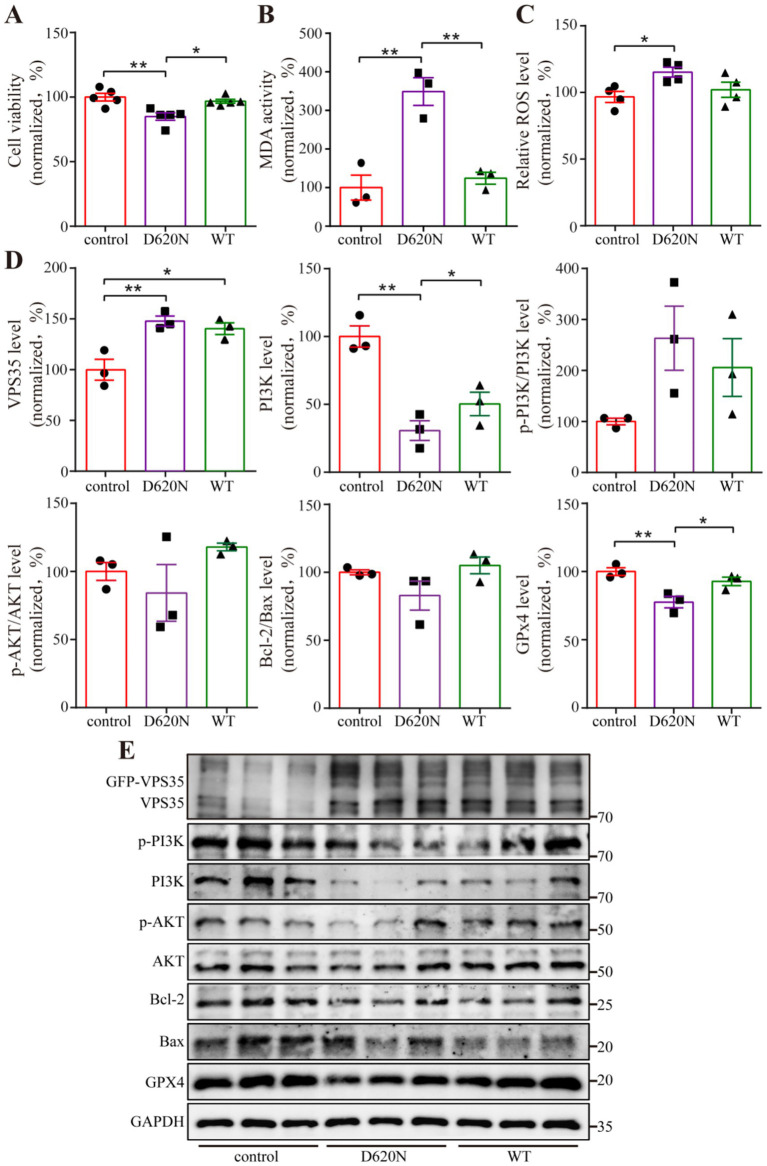
VPS35 D620N increases oxidative stress and ferroptosis. **(A)** N2a cells are transfected with empty vector (control), VPS35 wild-type (WT), and VPS35 D620N mutant (D620N) plasmids, and cell viability is detected by CCK-8 kit after 48 h transfection. **(B,C)** After 48 h of transfection, the level of lipid peroxidation and ROS is detected by a related kit. **(D,E)** Western blot detects the associated protein level, and statistical analysis was performed by using One-way ANOVA followed by Tukey’s post hoc. Values are shown as the mean ± SEM (*n* ≥ 3), *p < 0.05, **p < 0.01.

Molecular docking analysis revealed a strong binding affinity between VPS35 and the key target PI3K (subtype: PI3K3C3) and AKT1. To investigate the functional impact of the VPS35 D620N mutation on PI3K-Akt signaling and its regulatory role in proliferation and cell death, N2a cells were transfected with WT or D620N plasmids. CCK-8 assays found that the D620N mutation significantly suppressed cell viability compared to the WT and control groups (Figure 6A). Given the critical role of oxidative stress in PD pathogenesis, we further assessed lipid peroxidation and reactive oxygen species (ROS) levels, but the ratio of Bcl-2 to Bax, a related protein of apoptosis, had no significance among the three groups. The D620N group exhibited markedly elevated lipid peroxidation and ROS accumulation relative to both WT and control groups (Figures 6B,C), suggesting that D620N enhanced oxidative damage.

### 3.7 VPS35 D620N promotes ferroptosis via GPX4 suppression and PI3K-Akt pathway inhibition

Ferroptosis, a novel iron-dependent cell death pathway characterized by iron dysregulation and lipid peroxide accumulation, has been increasingly implicated in the pathogenesis of PD, where iron accumulation and oxidative stress drive dopaminergic neuron degeneration (Ding et al., 2023; Stockwell et al., 2017), and was subsequently evaluated. Western blot analysis revealed a significant reduction in glutathione peroxidase 4 (GPX4), a key ferroptosis suppressor, in D620N-transfected cells compared to WT and control groups. Concurrently, PI3K protein levels were downregulated in D620N-expressing cells, accompanied by a declining trend in downstream Akt pathway activity (Figures 6D,E). However, the VPS35 D620N mutation did not cause the statistically significant alterations of the ERK1/2 and phosphorylated ERK1/2 proteins level compared to control or VPS35 WT groups (Supplementary Figure 2).

## 4 Discussion

This study elucidated that the VPS35 D620N mutation inhibits neurogenesis and promotes cell death *in vitro* and *in vivo*. Bioinformatics identified 10 neurogenesis-related targets (TP53, AKT1, and SRC) linked to PI3K-Akt, cAMP, and MAPK pathways, and molecular docking confirmed that VPS35 has strong binding properties with the targets, especially PI3K and AKT1. Therefore, we focused on studying the effect of VPS35 D620N on the PI3K-Akt signal axis. Experimentally, D620N reduced cell viability while elevating lipid peroxidation and ROS. Mechanistically, it downregulated ferroptosis-related GPX4 and PI3K-AKT signaling proteins, highlighting their critical role in VPS35 D620N-mediated neurogenic regulation.

In the study, we demonstrated the impact of VPS35 D620N on neurogenesis *in vitro* and *in vivo*. Our findings clearly showed that there is a pronounced and statistically significant decrease in neurogenesis within both the NPCs *in vitro* and *vivo,* and conversely, cell death is increased, which is manifested by a notable decline in the proliferation of neural cells and the generation of new neurons. These results are in agreement with previous reports that have shown that the VPS35 D620N mutant mice inhibit the processes of neurogenesis by interfering with neurotransmission and interacting with amyloid precursor protein (APP) (Rowlands and Moore, 2024; Jiang et al., 2021). In this study, our acute overexpression cellular model is designed to reveal the intrinsic toxic potential of the VPS35 D620N mutant, and this rapid onset of phenotypes *in vitro* likely reflects an amplification of core pathogenic pathways in a simplified system lacking the compensatory mechanisms present *in vivo*. However, this accelerated model does not recapitulate the slow time course of human PD but rather uncovers the primary cellular vulnerabilities initiated by the mutation. Moreover, VPS35 is a critical component of the retromer complex, and its proper function is essential for cellular homeostasis (Huang et al., 2022; Zavodszky et al., 2014). The introduction of additional VPS35 (even the wild-type) may subtly perturb the delicate balance of the retromer complex. In response, the cell might initiate a compensatory mechanism to upregulate the expression of its endogenous, correctly regulated VPS35 in an attempt to maintain core retromer function.

To explore the molecular mechanisms underlying VPS35 D620N-mediated regulation of neurogenesis, we integrated bioinformatics and molecular docking approaches to identify potential targets linking VPS35, PD, and neurogenesis. Our analysis revealed 1,099 overlapping targets among these three categories, with PPI network analysis highlighting tumor protein 53 (TP53), alpha serine/threonine-protein kinase 1 (AKT1), and src tyrosine-protein kinase (SRC) (the top three hub targets) were identified as central nodes. Reported studies have underscored the crucial role of TP53 in maintaining neuronal integrity and genomic stability. TP53 achieves this by precisely regulating apoptosis, DNA repair mechanisms, and antioxidant stress responses, thereby exerting a profound influence on neuronal development and maturation (Kim et al., 2017; Galderisi et al., 2003). In the context of PD, the dysregulated activation of TP53 has been implicated in promoting neuronal apoptosis and oxidative stress, which in turn exacerbates the neurodegenerative processes (Szybinska and Lesniak, 2017; Wolfrum et al., 2022). AKT1 and SRC regulate neuronal growth, proliferation, differentiation, and synaptic transmission through a complex signaling network. Moreover, they have been firmly established as significant contributors to the pathogenesis of PD (Cheng et al., 2022; Portugal et al., 2022).

Furthermore, an in-depth analysis of the Kyoto Encyclopedia of Genes and Genomes (KEGG) pathways has unveiled the intricate signaling mechanisms that intricately link VPS35, PD, and neurogenesis. This signaling network encompasses several key elements, such as the phosphoinositide 3-kinase-alpha serine/threonine-protein kinase (PI3K-Akt) pathway, the cyclic adenosine monophosphate (cAMP) pathway, the Ras signaling cascade, and the mitogen-activated protein kinase (MAPK) pathway. In the context of PD, the PI3K-Akt signaling pathway has been demonstrated to possess neuroprotective properties by mitigating neurotoxicity, thus promoting the survival and maturation of neuronal populations in critical brain regions such as the hippocampus and substantia nigra pars compacta (Yang et al., 2021; Luo et al., 2019). For example, recent research has shown that enhancing the activity of the PI3K-Akt pathway can rescue neurons from apoptosis induced by various PD-related toxins (Goyal et al., 2023; Long et al., 2021). The cAMP signaling pathway is recognized for its significant role in sustaining neuronal viability and facilitating axonal outgrowth (Aglah et al., 2008; Mussen et al., 2023). Additionally, dysregulation of the Ras signaling pathway and its downstream kinases has been closely associated with the pathogenesis of neuronal degeneration and motor impairments characteristic of PD (Gravandi et al., 2023). Interestingly, modulating the activation of the MAPK pathway has emerged as a promising therapeutic strategy. Inhibition of the MAPK pathway has been shown to effectively attenuate oxidative stress (Cui et al., 2022), suppress inflammatory processes (Zhang et al., 2023), and alleviate the damage to dopaminergic neurons (Gravandi et al., 2023), thereby exerting a robust neuroprotective effect on PD mice. These pathways are essential for the regulation of neurogenesis in the pathophysiology of PD. Thus, in the adult hippocampus, PI3K-Akt, MAPK, and p53 signaling pathways control neural stem cell activation, progenitor proliferation, differentiation, and the removal of excess cells via programmed cell death (Sanchez-Castillo et al., 2022). In this study, the VPS35 D620N mutation disrupts cellular homeostasis and vesicular trafficking, impairing precise regulation of these pathways, resulting in reduced progenitor proliferation and differentiation, increased cell death susceptibility, and ultimately the loss of new neurons. These effects, though opposite to tumor cell proliferation, stem from similar molecular failures. Notably, the VPS35 D620N mutation interacts with multiple genes and pathways to impair neuronal function. For example, it downregulates AKT1, weakens PI3K-Akt survival signaling (Kang et al., 2014), disrupts autophagy and lysosomal degradation, activates TP53/p53 signaling, and induces neuronal apoptosis (Yang et al., 2020). Additionally, it also alters SRC, promotes α-synuclein accumulation, as well as exacerbates neuroinflammation (Nie et al., 2021) and disrupts Wnt/β-catenin, EGFR/Ras/MAPK, and MAPK1/3 signaling, reducing neurotrophic support and promoting protein aggregation (Kvainickas et al., 2017; Huang et al., 2022). Enhanced HDAC1 activity via mTORC1 suppresses synaptic gene expression and disrupts synaptic plasticity, ultimately damaging neuronal survival, protein homeostasis, and synaptic function (Taylor et al., 2024). In addition, we found that the VPS35 D620N mutant leads to the protein level alteration of the PI3K-Akt pathway, but no significant change in the MAPK pathway associated with protein level. Combined with molecular docking technology, data demonstrated robust binding affinities between VPS35 and PI3K. We supposed that the PI3K-Akt pathway is a potential role for VPS35-modulated neurogenesis in PD.

The interaction between VPS35 and the PI3K/AKT1 axis is particularly intriguing, although direct evidence specifically documenting this interaction remains limited in current reports. Our study reveals that the VPS35 D620N mutation not only significantly impairs cell viability but also triggers an increase in lipid peroxidation and the production of ROS. Given that ferroptosis is characterized by elevated lipid peroxidation and ROS levels, we focused on detecting the key ferroptosis-related protein GPX4. Intriguingly, our results showed that the D620N mutation leads to a notable decrease in the level of GPX4 and has no significant effect on the ratio of apoptotic-related proteins Bcl-2 and Bax. Additionally, we observed a downward trend in the levels of proteins associated with the PI3K-Akt signaling pathway. These data are consistent with previous research that has associated VPS35 deficiency with abnormal AKT activation and mitochondrial dysfunction in PD models (Roque et al., 2022; Tang et al., 2015; Zhou et al., 2017). The VPS35 D620N mutant leads to a decrease in PI3K protein levels. Based on the known functions of VPS35 in intracellular transport and protein degradation, we speculate that the VPS35-PI3K interaction may redirect PI3K to the lysosomal pathway, promoting its degradation. This interaction may alter the stability of PI3K or its binding to larger protein complexes, making it more easily degraded by proteasomes, and D620N mutations may exacerbate this process. Our findings suggested that the VPS35 D620N mutation may exacerbate ferroptosis susceptibility through GPX4 depletion and PI3K-Akt signaling attenuation, thereby impairing downstream neurogenic processes. The expression of the D620N mutant disrupts the fundamental cellular homeostasis in neural progenitor cells, which may manifest as impaired growth factor signaling due to incorrect receptor sorting, leading to weakened pro-neurogenic signals (Jiang et al., 2021). Additionally, mitochondrial dysfunction causes energy stress, depriving the cell of the energy required for division and differentiation (Hanss et al., 2021). Accumulation of cellular damage, due to impaired autophagy-lysosomal clearance, results in a general state of cellular distress (Shiraishi et al., 2024). Under such stress, neural progenitor cells may fail to proliferate effectively, initiate differentiation programs, or be driven toward apoptosis or cell cycle arrest. Consequently, the GFP-positive/D620N-expressing cells create a compromised local microenvironment that indirectly prevents neighboring progenitor cells (which may be GFP-negative but are in the same niche) from successfully incorporating EdU and proceeding with neurogenesis. Notably, while our study has shed light on these associations, the specific and intricate regulatory mechanisms governing how the VPS35 D620N mutation influences ferroptosis via GPX4 and the PI3K-Akt pathway, and subsequently impacts neurogenesis, remain largely elusive. Although our MDS provided a computationally robust prediction, demonstrating a high binding affinity and stable complex formation between VPS35 and PI3K, and *in silico* evidence offers a compelling structural model for a direct interaction, its biological validation *in vivo* remains to be established. Confirming this putative interaction experimentally represents a crucial next step in elucidating the mechanistic basis of VPS35-related pathology. In addition, future investigations utilizing transgenic VPS35 D620N mouse models will be essential to directly quantify the reduction of PI3K *in vivo* and to solidify its contribution to the observed neurodegenerative phenotypes. Therefore, additional in-depth experimental investigations are essential to fully elucidate these complex mechanisms and to gain a comprehensive understanding of their implications for neuronal development and the pathogenesis of related neurological disorders.

## 5 Conclusion

In summary, this study uses experiments, bioinformatics, and molecular docking to explore the impact and potential mechanisms of VPS35 D620N on neurogenesis in PD models. Our findings indicate that the VPS35 D620N mutation inhibits neurogenesis in PD, likely through hub targets such as PI3K and AKT1, and the PI3K-Akt signaling pathway, providing mechanistic insights into its role in PD-associated neuronal degeneration.

## Data Availability

The original contributions presented in the study are included in the article/Supplementary material, further inquiries can be directed to the corresponding author.

## Footnotes

^1^ https://www.genecards.org/

^2^ https://bioinfogp.cnb.csic.es/tools/venny/index.html

^3^ https://string-db.org

^4^ http://www.bioinformatics.com.cn/

^5^ https://www.rcsb.org/search

^6^ https://www.ncbi.nlm.nih.gov/pubmed/

^7^ https://gramm.compbio.ku.edu/
