# Supplementary material for: VPS35 D620N mutation impairs neurogenesis and promotes ferroptosis in Parkinson’s disease by using molecular docking, molecular dynamic simulation, and cellular model
Source: Front Aging Neurosci. 2025 Nov 25;17:1692687. doi: 10.3389/fnagi.2025.1692687 (PMC12685849; doi:10.3389/fnagi.2025.1692687)
Supplement: Supplementary file 1 [file Table_1.docx]

**VPS35 D620N mutation impairs neurogenesis and promotes ferroptosis in Parkinson’s disease via using molecular docking, molecular dynamic simulation and cellular model**

Mei Jiang^1,2^, Xu Deng^1,#^, Zijie Qiu^1,#^,Yuan Fu^1^, Zixiong Qiu^1^, Jiankai Zhang^1^, Hongxia Fu^2^, Jie Li^1^, Yao Luo^1^, Xiaojun Cui^1,*^

1.The Affiliated Dongguan Songshan Lake Central Hospital, Guangdong Medical University, Dongguan 523326, China. Dongguan Key Laboratory of Stem Cell and Regenerative Tissue Engineering, Department of Human Anatomy, School of Basic Medicine, Dongguan Campus, Guangdong Medical University, Dongguan 523808, China.

2.The Affiliated Dongguan Songshan Lake Central Hospital, Guangdong Medical University, Dongguan 523326, China.

^#^These authors contributed equally to this work.

***Corresponding authors:**

Xiaojun Cui: cuixiaojun@gdmu.edu.cn**Supplementary figures**

**
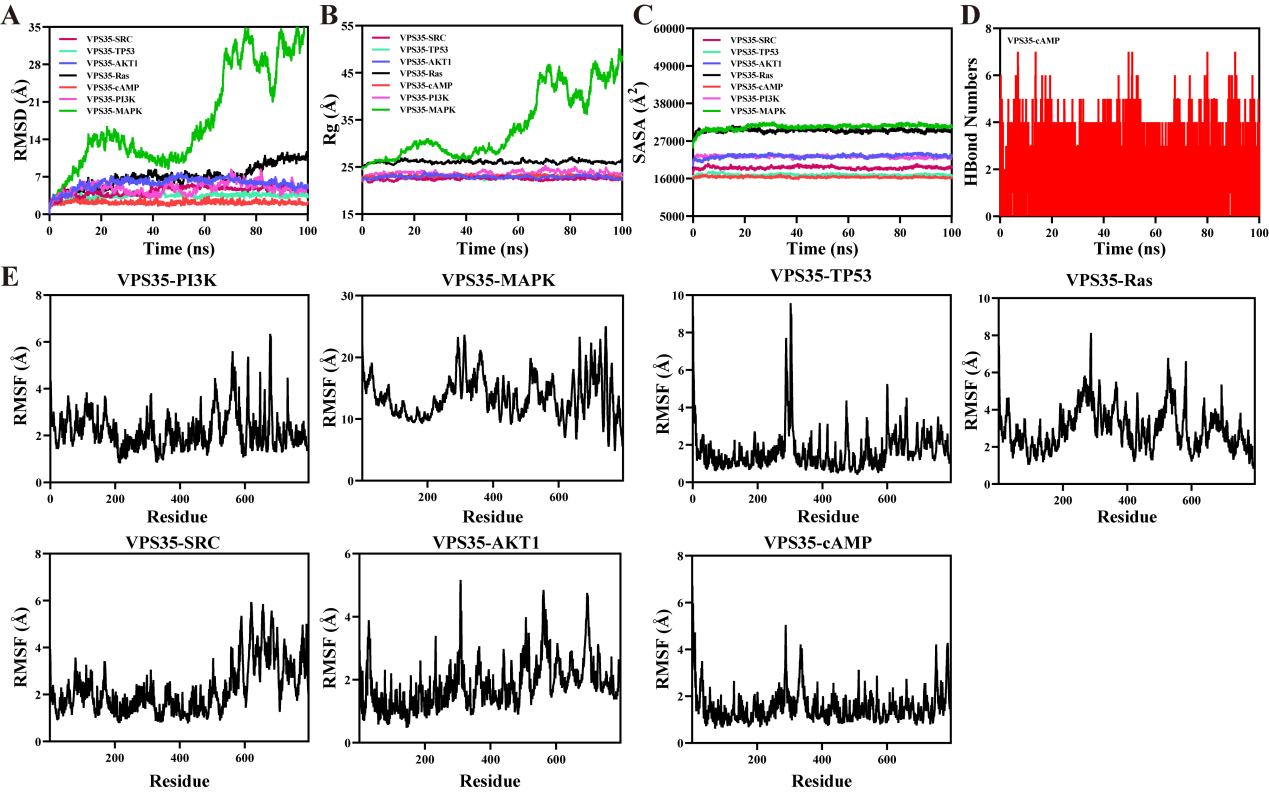
**

**sFigure 1. The interaction diagram between VPS35 WT and key targets by using molecular dynamics simulations.** (A) Time-dependent RMSD values of protein-ligand complexes. (B) Time-dependent Rg values of protein-ligand complexes. (C) Time-dependent SASA values of protein-ligand complexes. (D) Time-dependent HBonds values of protein-ligand complexes. (E) RMSF values of protein-ligand complexes.


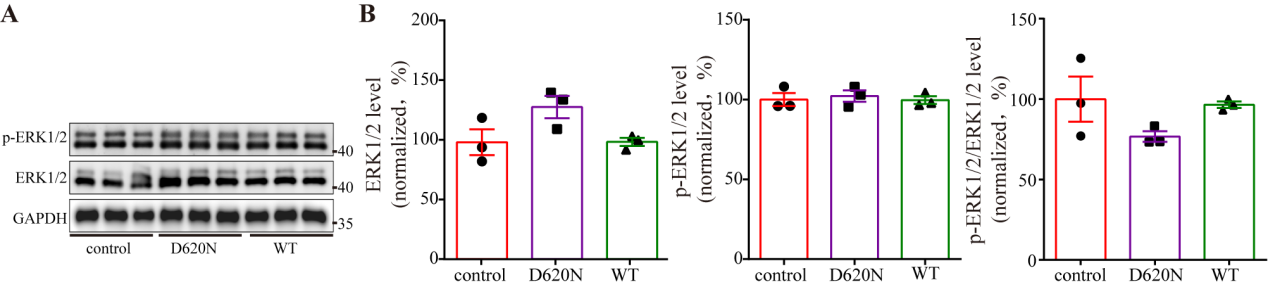


**sFigure 2. VPS35 D620N didn’t change the MAPK pathway associated ERK1/2 protein level.** (A-B) After 48h transfection, Western blotting detects the associated protein level and statistic analysis were performed by using One-way ANOVA followed by Tukey’s post hoc. Values are shown as the mean±SEM (n=3).
